# *Oxalisxishuiensis* (Oxalidaceae), a new species from Danxia landforms in Guizhou, China

**DOI:** 10.3897/phytokeys.239.119046

**Published:** 2024-03-27

**Authors:** Yan-Bing Yang, He Li, Ming-Tai An, Lang Huang, Guo-Xiong Hu, Cheng-Hua Yang, Zheng-Xian Dai

**Affiliations:** 1 Key Laboratory of National Forestry and Grassland Administration on Biodiversity Conservation in Karst Mountainous Areas of South-western China, Guizhou Academy of Forestry, Guiyang 55005, Guizhou, China; 2 Fanjingshan National Observation and Research Station of Chinese Forest Ecosystem, Jiangkou 554400, Guizhou, China; 3 College of Forestry, Guizhou University, Guiyang 550025, Guizhou, China; 4 Center for Biodiversity and Natural Conservation, Guizhou University, Guiyang 550025, Guizhou, China; 5 College of Life Science, Guizhou University, Guiyang 550025, Guizhou, China; 6 Guizhou Xishui Nature Reserve Management Bureau, Xishui 564600, Guizhou, China

**Keywords:** China, Danxia landforms, Oxalidaceae, *Oxalis*, Xishui

## Abstract

*Oxalisxishuiensis*, a new species of Oxalidaceae from Danxia landforms of Xishui County, Guizhou, China, is described and illustrated. It is morphologically similar to *O.wulingensis* by the two lateral leaflets arranged at about 180° angle and oblong pink petals with lilac veins, but clearly differs from the latter by leaflets almost as long as wide, obliquely obcordate lateral leaflets, shorter peduncles, longer capsule (1.2–1.5 cm vs. 0.5–0.7 cm) and alveolate seeds.

## ﻿Introduction

*Oxalis* L. contains about 500–800 species and is distributed all over the world, but South America and southern Africa are thought to be the two centres of diversity (Azkue 2000; Sidwell and Knapp 2002; Vaio et al. 2016). This genus is characterised by 3-foliolate leaves, solitary, cymose or umbellate inflorescence, five-numerous flowers, free sepals and petals (Liu and Watson 2008). Based on morphological characteristics, the genus was divided into Oxalissubgen.Thamnoxys (Endl.) Reiche, O.subgen.Monoxalis (Small) Lourt., O.subgen.Oxalis L. and O.subgen.Trifidus Lourt. (Moura et al. 2020). In the *Flora of China*, six native species in *Oxalis* are recorded, namely *O.acetosella* L., *O.corniculata* L., *O.griffithii* Edgew. & Hook. f., *O.leucolepis* Diels, *O.obtriangulata* Maxim. and *O.stricta* L. (Liu and Watson 2008). In the past two decades, two new species were described in China (*O.wulingensis* T. Deng, D. G. Zhang & Z. L. Nie and *O.shibeishanensis* Huan C. Wang & Y. Tian) (Deng et al. 2013; Tian et al. 2020).

During field surveys to Xishui County, north Guizhou Province, China, in November 2022, a population of *Oxalis* with special morphological characteristics attracted our attention. To conduct further detailed observation, we transplanted five individuals in the greenhouse of the Guizhou Academy of Forestry and three individuals were made into herbarium specimen after flowering. After careful morphological examination and comparison with morphologically similar species in *Oxalis*, it is confirmed as an undescribed new species of *Oxalis*. Here, we formally describe this new species.

## Materials and methods

Morphological characteristics were observed and measured from the living plants. The comparison with morphologically similar species was based on the digital specimens from the online database CVH (https://www.cvh.ac.cn/) and JSTOR Global Plants (https://plants.jstor.org/), as well as the descriptions from relevant literature (Liu and Watson 2008; Deng et al. 2013; Aoki et al. 2019; Tian et al. 2020).

## Taxonomic treatment

### Oxalis xishuiensis

Taxon classificationPlantaeOxalidalesOxalidaceae

Y.B. Yang, M.T. An & H. Li sp. nov.

A0D39500-2473-5BBB-B214-E6F488D93FC6

urn:lsid:ipni.org:names:77339170-1

Figs 1 , 2

#### Type.

China. Guizhou Province, Xishui County, Xishui National Nature Reserve, 28°8'25"N, 105°53'32"E, alt. 1200 m, 10 November 2022, xs2022103 (holotype: GF, isotypes: GZAC).

**Figure 1.**
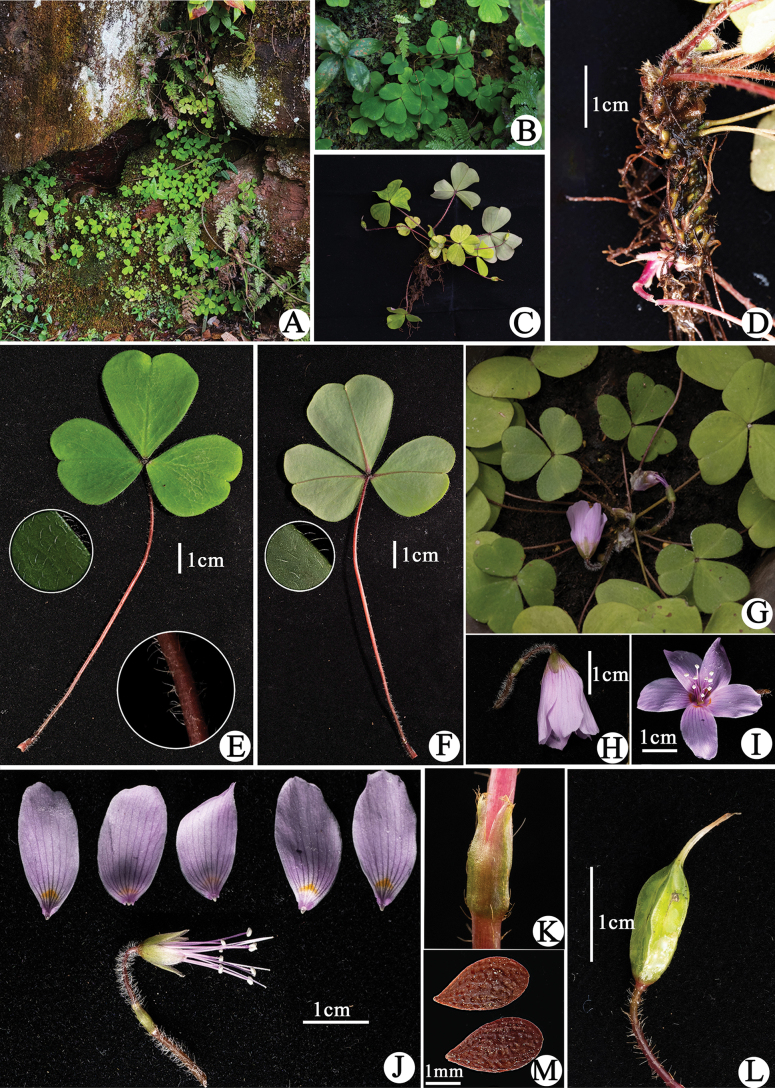
*Oxalisxishuiensis* Y.B. Yang, M.T. An & H. Li **A, B** habitat **C** plants **D** rhizome **E** upper surface of leaves **F** lower surface of leaves **G** flowering plant **H** lateral view of the flower **I** frontal view of the flower **J** dissected flower **K** bract **L** capsule **M** seeds.

#### Diagnosis.

The new species is most morphologically similar to *Oxaliswulingensis*, but differs from the latter by its leaf blade ca. as long as wide, obliquely obcordate lateral leaflets (vs. long obtriangular), shorter peduncle (ca. 3–4 cm long, shorter than leaves vs. 10–12 cm long, longer than leaves), longer capsule (1.2–1.5 cm long vs. 0.5–0.7 cm long) and alveolate seeds (vs. only with longitudinally ridge).

**Figure 2.**
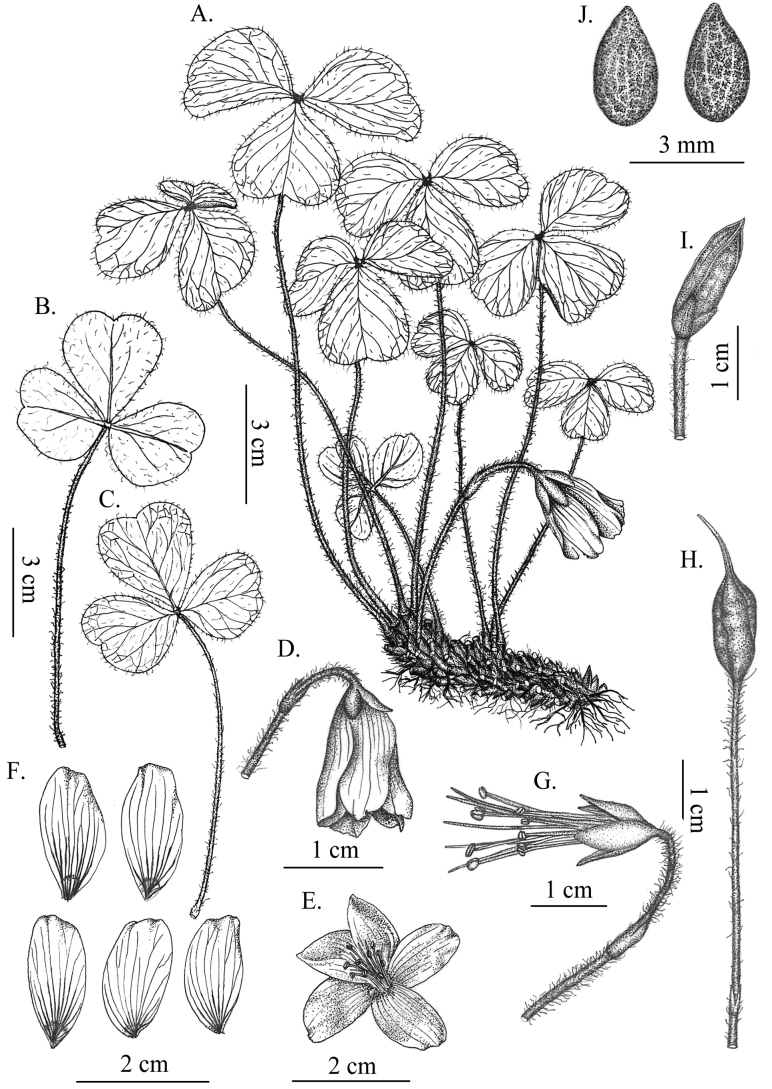
*Oxalisxishuiensis* Y.B. Yang, M.T. An & H. Li **A** habit **B** lower surface of leaves **C** upper surface of leaves **D** lateral view of the flower **E** frontal view of the flower **F** petals **G** stamens **H** chasmogamous capsule **I** cleistogamous capsule **J** seeds.

#### Description.

Perennial herbs, 8–15 cm tall; rhizome creeping underground, densely covered by dark brown, scale-like remains of leaf bases, ca. 1 cm thick including scales; scales pilose. Leaves radical, 3-foliolate, the two lateral leaflets arranged at about 180° angle; petioles 4.5–8 cm long, densely covered with white, pubescent over their entire length; lateral leaflets blades obliquely obcordate, 1.4–2.1 × 1.3–2 cm; middle leaflet blades obcordate, 2–3.1 × 1.9–3 cm; leaflets blades adaxially light green to green, abaxially pale green, purple when young; both surfaces white pubescent or adaxially glabrous, apex broadly emarginate, base cuneiform, lobe apices obtuse. Flowers solitary, nodding; peduncles ca. 3–4 cm long, shorter than leaves at flowering time, peduncle much longer than petioles due to pedicel extension at maturity of capsule; bracts at middle of flowering stalk, triangular, ca. 4 mm long, apex bifid with dense trichomes along mid-vein and margins; sepal oblong, ca. 7 × 3 mm, green, surface and margins with some hairs, persistent; petals pink with lilac veins and a yellow patch at base, oblong, ca. 2 × 1 cm, apex obtuse or irregularly denticulate; stamens 10, alternately long and short, the longer ones ca. 1.8 cm, the shorter ones ca. 1.4 cm, all basally connate, filaments purple-red, glabrous, anthers white; pistil ca. 2.1 cm long; ovary glabrous, locules 5, each with a single ovule, styles 5, slender, stigma linear. Capsule erect, cleistogamous capsule with persistent calyx, ovoid to oblong 1.2–1.5 × 0.4–0.5 cm, with five alar ridges; seeds ovoid, ca. 3 × 2 mm, with longitudinally ridge and alveolate on surfaces, dark brown when dry.

#### Distribution and habitat.

*Oxalisxishuiensis* is currently only known from Danxia landforms hills in the Xishui National Nature Reserve, Xishui County, Guizhou Province, south-western China. It grows on humid slopes in purple sand shale under the evergreen broad-leaved forest, at an altitude of 1200 m, along with *Marchantiapolymorpha* L., *Pteriscretica* L., *Metathelypterislaxa* (Franch. & Sav.) Ching, *Trigonotisomeiensis* Matsuda, *Saxifragastolonifera* Curtis, *Carexbaccans* Nees and *Lysimachiaparidiformis* Franch.

#### Phenology.

Chasmogamous flowers from February to March; Cleistogamous flowers from May to June. Fruiting from February to July.

#### Etymology.

The species epithet, *xishuiensis*, refers to the type locality of the new species.

#### Vernacular name.

习水酢浆草 (xí shuǐ cù jiāng cǎo)

#### Conservation status.

Currently, only one population of the new species with approximately 60 individuals has been found. Danxia landforms are widely distributed in this area, so we speculate that there may be other populations of this new species. Due to insufficient field investigations, the natural range of this species in the wild is unclear. According to the IUCN Red List Categories and Criteria (IUCN 2022), we recommend this species placement in the ‘Data Deficient’ (DD).

## Discussion

In the *Oxalis*, there are eight native species in China, but only two native species in Guizhou (Liu and Watson 2008; Deng et al. 2013; Tian et al. 2020). Discovery of *Oxalisxishuiensis* adds to the native local floras.

According to the classifications by Lourteig (2000) and Aoki et al. (2019), *Oxalisxishuiensis* should be classified into Oxalissubgen.Oxalis sect. Oxalissubsect.Oxalis. *Oxalisxishuiensis* is characterised by the obliquely obcordate lateral leaflets arranged at about 180° angle, shorter peduncles and alveolate seeds. This unique combination of morphological characteristics distinguishes *O.xishuiensis* from all other species of subsect. Oxalis (Liu and Watson 2008; Deng et al. 2013; Aoki et al. 2019; Tian et al. 2020). We made a detailed morphological comparison between *O.xishuiensis* and its relatives (Table 1). *Oxalisxishuiensis* morphologically is most similar to *O.wulingensis* by the two lateral leaflets arranged at about 180° angle and oblong pink petals with lilac veins, whereas *O.xishuiensis* leaflets are almost equal in length and width, two lateral leaflet blade shapes are asymmetric (obliquely obcordate) and smaller in size than the leaflet blades in the middle, mature leaf blades abaxially pale green (vs. purple in *O.wulingensis*), peduncles shorter than leaves, longer capsule 1.2–1.5 cm long (vs. 0.5–0.7 cm in *O.wulingensis*) and seeds with alveolate on both surfaces. Furthermore, the new species was discovered only from Danxia landforms hills, which is completely different from *O.wulingensis* growing in limestone habitat. *Oxalisxishuiensis* resembles *O.acetosella* in the obcordate leaf blades, but differs in leaf blades ca. as long as wide, two lateral leaf blades asymmetric and arranged at about 180° angle, peduncles shorter than leaves and petals oblong, pink with lilac veins (vs. obovate, white, lilac to pinkish veined in *O.acetosella*).

**Table 1.** Morphological comparison of species of *Oxalisxishuiensis* and its relatives.

Characters	*O.xishuiensis*	*O.wulingensis*	*O.acetosella*	*O.griffithii*
Rhizomes (including scales)	Ca. 10 mm in diameter	Ca. 10 mm in diameter	Ca. 3 mm in diameter	6–12 mm in diameter
Two lateral leaflets arrangement	About 180° angle	180° angle	120° angle	120° angle
Leaflets	Two lateral leaflets obliquely obcordate, 1.4 –2.1 × 1.3–2 cm; middle leaflets obcordate, 2–3.1 × 1.9–3 cm	Long obtriangular, 2.2–3.1 × 1.6–2.5 cm	Obcordate, 0.5–2 × 0.8–3 cm	Obtriangular, 1–2.5(–4.5) × 1.5–3.5(–5.5) cm
Leaflet apex	Broadly emarginate	Broadly emarginate	Deeply emarginate	Broadly emarginate to subtruncate
Leaflet indumentum	Both surfaces white pubescent or adaxially glabrous	Both surfaces villous (densely covered with long, brown hairs)	Both surfaces pubescent	Abaxially pubescent, adaxially glabrous
Leaflet adaxial surface colour	Light green to green	Green	Green	Green
Leaflet abaxial surface colour	Pale green, purple when young	Purple	Whitish-green, purple or red	Pale green or green
Peduncles in flowering	Ca. 3–4 cm long, shorter than leaves	10–12 cm long, longer than leaves	Peduncle equal to or longer than leaves	4–15 cm long, equal to or longer than leaves
Petal	Oblong ca. 2 cm, apex obtuse or irregularly denticulate	Oblong ca. 2.5 cm, apex obtuse or irregularly denticulate	Obovate, (1.2–)1.5–2.2 cm, apex retuse to deeply emarginate	Narrowly obovate, 1.2–1.6 (–2) cm, apex retuse to deeply emarginate
Petal colour	Pink with lilac veins	Pink with lilac veins	White, lilac to pinkish veined	White, rarely pink (Hubei)
Capsules	Ovoid or oblong, 12–15 × 4–5 mm	Ovoid, 5–7 mm long	Ovoid, 3–4 mm	Oblong-conic, 5–13 × 5–6 mm
Seed	Ovoid, ca. 3 mm long, with longitudinally ridge and alveolate	Ovoid, ca. 2.1 mm long, with longitudinally ridge	Ovoid, with longitudinally ridged	Ovoid, 2.5–3.5 mm, with longitudinally ridged
Flowering time	Chasmogamous flowers: February to March, Cleistogamous flowers: May to June	March	July to August	March to September

## Supplementary Material

XML Treatment for Oxalis xishuiensis

## ﻿Additional information

### Author ORCIDs

Yan-Bing Yang https://orcid.org/0009-0007-9014-1817

He Li https://orcid.org/0009-0006-0597-3209

Ming-Tai An https://orcid.org/0000-0003-3886-0287

Lang Huang https://orcid.org/0009-0004-8038-7432

Guo-Xiong Hu https://orcid.org/0009-0007-6983-0626

Cheng-Hua Yang https://orcid.org/0000-0002-5941-676X

Zheng-Xian Dai https://orcid.org/0009-0003-7938-7243

### Data availability

All of the data that support the findings of this study are available in the main text.
